# The application effect of the Rasch measurement model combined with the CRF model: An analysis based on English discourse

**DOI:** 10.1371/journal.pone.0309001

**Published:** 2024-08-22

**Authors:** Yunxia Wang

**Affiliations:** Nanyang Medical College, Nanyang, Henan, China; Air Force Engineering University, CHINA

## Abstract

To analyze English discourse more accurately and provide more detailed feedback information, this study applies Rasch measurement and Conditional Random Field (CRF) models to English discourse analysis. The Rasch measurement model is widely used to evaluate and quantify the potential traits of individuals, and it has remarkable advantages in measurement and evaluation. By combining the CRF model, the Rasch model is employed to model the structural and semantic information in the discourse and use this model to carry out sequence labeling, to enhance the ability to capture the internal relations of the discourse. Finally, this study conducts comparative experiments on integrating the Rasch measurement and CRF models, comparing the outcomes against traditional scoring methods and the standalone CRF model. The research findings indicate that: (1) The discourse component syntactic analysis model on the Penn Treebank (PTB) database obtained Unlabeled Attachment Score (UAS) values of 94.07, 95.76, 95.67, and 95.43, and Labeled Attachment Score (LAS) values of 92.47, 92.33, 92.49, and 92.46 for the ***L***_***OC***_, ***C***_***RF***_, ***C***_***RF***_**2**_***O***_, and ***M***_***FVI***_ models, respectively. After adding the Rasch measurement model, the UAS values of the four models on the PTB database are 96.85, 96.77, 96.92, and 96.78 for the ***L***_***OC***_, ***C***_***RF***_, ***C***_***RF***_**2**_***O***_, and ***M***_***FVI***_ models, respectively, with LAS values of 95.33, 95.34, 95.39, and 95.32, all showing significant improvement. (2) By combining contextual information with CRF models, students can better understand their discourse expression, capture the connections between English discourse sentences, and analyze English discourse more comprehensively. This study provides new ideas and methods for researchers in English language education and linguistics.

## Introduction

With the acceleration of globalization, the importance of teaching and researching English as the primary language for international communication is becoming increasingly evident. In assessing English language proficiency, how to scientifically and accurately measure learners’ language levels has become a crucial issue in educational research and practice. In recent years, the Rasch measurement model and the Conditional Random Field (CRF) model, as two advanced statistical models, have shown great potential in fields such as educational assessment and natural language processing (NLP) [1]. This study explores the combined application of these two models in English text analysis, aiming to offer new ideas and methods for English language proficiency assessment.

The Rasch model, proposed by Danish mathematician Georg Rasch in 1960, is a probabilistic Item Response Theory (IRT) model. Based on the theory of latent variables, the model quantifies individual abilities and item difficulties to achieve objective and interval measurements. In the Rasch model, individual abilities and item difficulties are quantified through ability parameters and difficulty parameters. In addition, it is assumed that there is an "equivalence" between the two, meaning that when ability matches difficulty, the probability is approximately 50%. This characteristic gives the Rasch model high comparability and reliability in assessing individual abilities and item difficulties [2]. In the field of education, the Rasch model has been extensively used for student ability assessments, quality testing of subject exams, learning style diagnostics, and more, providing the scientific basis and technical support for education and teaching. CRF, proposed by Lafferty in 2001 based on the maximum entropy model and the Hidden Markov Model (HMM), is a discriminative probabilistic undirected graphical model [3]. CRF is mainly used for sequence labeling problems, learning the relationship between input sequences and corresponding label sequences to make predictions and annotations. In NLP, CRF has exhibited high accuracy and robustness in tasks such as part-of-speech tagging, named entity recognition, and word segmentation due to its ability to consider contextual information and global consistency. Additionally, the flexibility and extensibility of CRF enable it to handle more complex tasks, such as semantic role labeling and named entity relationship extraction.

The main contributions of this study are as follows: (1) Combining the Rasch measurement model and the CRF model: This study innovatively explores the Rasch measurement and CRF models’ integrated application in the syntactic analysis of English texts. By combining these two models, the study proposes a new method to handle the complex structures and semantic information within texts, thereby significantly enhancing the accuracy and efficiency of syntactic analysis. (2) Experiments prove the combined model’s effectiveness: Through detailed experimental comparisons on datasets, this study demonstrates that the combined syntactic analysis method using the Rasch measurement model and the CRF model outperforms traditional models on multiple metrics. Specifically, the combined model significantly improves evaluation metrics such as Unlabeled Attachment Score (UAS) and Labeled Attachment Score (LAS), validating its superiority in capturing syntactic structures and semantic information. (3) Performance improvement and application prospects: The research results indicate that the combined model achieves notable improvements in accuracy and recall in both English and Chinese text analysis tasks. This improves syntactic analysis in NLP applications and furnishes new methods and ideas for further research and application.

## Literature review

In the realm of CRF modeling, numerous researchers have extensively explored its applications. Wang et al. (2023) introduced a novel approach based on bidirectional gated recursive units for aircraft target intention recognition, incorporating a spatiotemporal attention mechanism using CRF. Their study provided a detailed analysis of aerial target intention recognition challenges, emphasizing crucial aspects of feature extraction and temporal alignment to meet specific interpretive requirements [4]. Geng (2022) proposed an innovative Structured Learning (SL) approach termed SLCRF. This method leveraged a three-dimensional convolutional autoencoder (3DCAE) to eliminate redundant pixel information and utilized spectral-spatial relationships between adjacent pixels. An iterative algorithm was developed to optimize the objective function of SLCRF. The proposed method’s effectiveness was rigorously evaluated on a challenging public hyperspectral imaging (HIS) dataset, demonstrating significant advancements in performance [5]. Furthermore, numerous studies have utilized the Rasch measurement model as a primary analytical tool to assess the effectiveness and reliability of measurement instruments across various domains. Initially, Mokshein et al. (2019) focused on evaluating sixth-grade English test papers, determining the difficulty levels of 40 multiple-choice questions. Employing the Rasch model, the researchers illustrated the test’s advantages in terms of reliability, validity, and item characteristics. The model analysis also identified issues such as item mismatch and interference factors, offering crucial insights for enhancing the assessment tool [6]. Subsequently, Dewi et al. (2023) conducted an item analysis of TOEFL reading comprehension questions to enhance test quality. Through Rasch modeling, they thoroughly analyzed 20 questions, identifying problematic items and providing specific recommendations for improvement, highlighting the Rasch model’s role in assessing item quality [7]. Fleary et al. (2022) developed and validated a test-based tool for assessing adolescent health literacy. Utilizing the Rasch model, they successfully crafted an effective assessment tool and substantiated its reliability and efficacy in measuring adolescent health literacy [8]. Wolfs et al. (2023) endeavored to develop and validate a test tool for assessing children’s musical abilities. Applying the Rasch model, they adeptly analyzed implicit pitch ability tests, confirming the test’s effectiveness and reliability in assessing children’s musical aptitude [9]. Finally, Roberts et al. (2022) validated the effectiveness and reliability of the Malaysian English textbook assessment checklist. Using the Rasch model, they convincingly demonstrated the checklist’s high validity and reliability, emphasizing its role in evaluating textbook quality [10].

Li et al. (2020) investigated the problem of identifying Elementary Discourse Units (EDUs). Based on a Bidirectional Long Short-Term Memory—Conditional Random Field (Bi-LSTM-CRF) model, they constructed a parallel Chinese-English text corpus and annotated and aligned EDUs. The model combined word embeddings, part-of-speech, and syntactic features, leveraging the advantages of Bi-LSTM and CRF to improve EDU identification. Experimental results showed that this method’s F1 score was about 2% higher than traditional methods, achieving satisfactory results in Chinese and English EDU identification. Feature contribution experiments indicated that combining all features could yield the best results, with syntactic features outperforming other features. This study offered new ideas and methods for cross-linguistic discourse unit identification, contributing to the accuracy and efficiency of text analysis [11]. Aryadoust et al. (2021) conducted a comprehensive review of the application of the Rasch measurement model in language assessment. They analyzed 215 papers from 21 applied linguistics journals and identified the use of 7 Rasch models and 23 software packages in these studies. The many-faceted Rasch model and Facets were the most commonly used models and software. The review highlighted prominent differences in the number of papers across different language skills and components, with writing and grammar being the most and least frequently investigated areas, respectively. Despite the increasing application of Rasch measurement in language assessment, many studies were found to inadequately report on critical aspects such as unidimensionality and local independence. Based on a multilayer network analysis, the study revealed two major discrete practice communities and provided guidelines and recommendations on unidimensionality, local independence, data-to-model fit, and reliability. These findings offered important references for further optimizing the use of Rasch measurement in language assessment [12]. Tian et al. (2021) explored the issues of adversarial attacks and defenses in drones using Deep Learning (DL) technology. Although DL technology improved performance in Cyber-Physical Systems (CPSs), it also introduced new security concerns. The study proposed two adversarial attack methods against DL-based drones, demonstrating how these methods created imperceptible adversarial images that posed threats to drone navigation and control. To address these challenges, the study evaluated adversarial training and defense distillation methods to improve the robustness of DL models in drones. This is the first systematic study on adversarial attacks and defenses in DL-based drones, emphasizing the necessity of ensuring the security of DL models in safety-critical applications [13].

In summary, the study by Li et al. demonstrated the Bi-LSTM-CRF model’s effectiveness in identifying discourse units in both Chinese and English texts. Aryadoust et al. revealed the widespread application and existing issues of Rasch measurement in language assessment. Meanwhile, Tian et al. explored adversarial attacks and defenses in drones using DL. These studies collectively highlighted the significant impact of model selection and feature utilization on the outcomes in language assessment and DL applications. This can provide a critical reference for the combined model’s application in English text analysis in the present study.

## Discourse analysis algorithm based on CRF

### The CRF model

**Fig 1:**
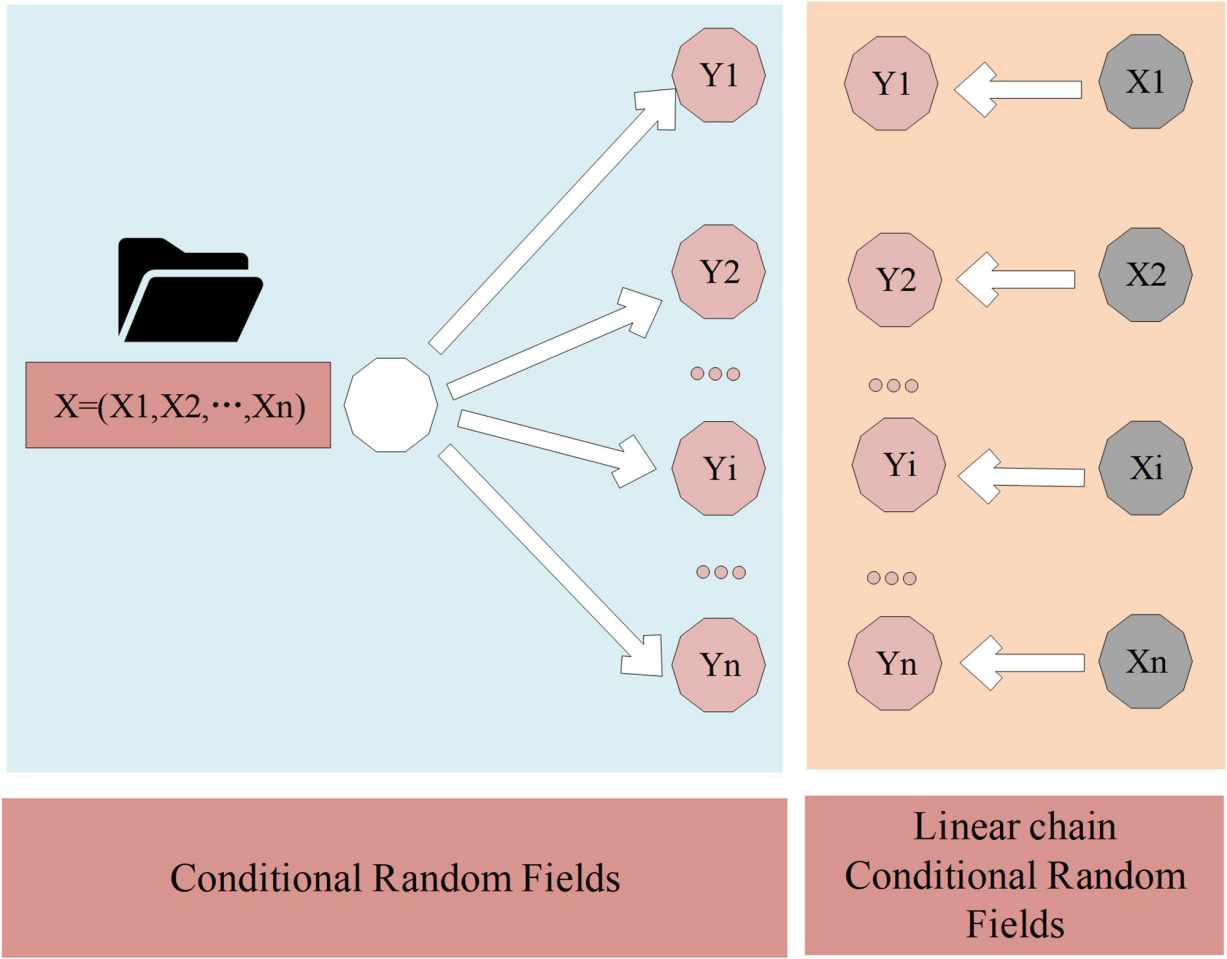
CRF structure.

In the task of English discourse analysis, CRF is used to model the conditional probability distribution of another set of output sequences Y given a set of input sequences X. In this task, X represents the sequence of sentences to be segmented, while Y refers to the segmentation results. A random field (RF) is a graphical model consisting of multiple nodes (random variables) connected by edges (dependencies). In RF, node assignments are randomly determined according to some distribution. When each node’s assignment depends only on its adjacent nodes, it is known as a Markov Random Field (MRF). CRF is a specific type of MRF where the model involves two variables: the input sequence X and the output sequence Y [14]. In CRF, Y forms an MRF, while X does not possess Markov properties, implying that event occurrences in the random process depend solely on their preceding events. Mathematically, CRF is described as follows: assuming X and Y are random variables, P(Y|X) represents the conditional probability distribution of Y given X. When the random variable Y forms an MRF, this conditional distribution P(Y|X) is referred to as CRF. Its structure is presented in Fig 1.

In the definition of CRF, there is no restriction on the structure of the input sequence X and the output sequence Y. However, in segmentation tasks where each character is annotated, the structure of the input sequence (sentence) corresponds directly to the structure of the output sequence (character annotations). This specific scenario is termed Linear Chain Conditional Random Fields (Linear-CRF). Feature functions can be categorized into two types: state features, defined on the Y nodes, which solely depend on the current node. They are represented by Eq (1):

$$S_{l}\left(y_{i},x,i\right),l=1,2,\ldots ,L$$ (1)

***L*** represents the number of feature functions defined at this node; ***i*** refers to the current node’s position in the sequence [15]; The other category is the transfer feature, which is a feature function defined on the edge. Therefore, this feature function is relevant only to the current and its previous node, as expressed in Eq (2):

$$t_{k}\left(y_{i-1}y_{i},x,i\right),k=1,2,\ldots ,K$$ (2)

The definition of ***K*** is the same as ***L***; the definition of ***i*** is the same as its definition in the state feature. The value of these two types of eigenfunctions is 0 or 1, namely, whether the eigenconditions are met [16]. Moreover, each feature function is weighted to express the trust of this feature function. Let the weight coefficient of the ***t***_***k***_ be ***λ***_***k***_, and the weight coefficient of the ***s***_***l***_ be ***μ***_***l***_, the above four parameters determine Linear-CRF, which can be written as:

$$P\left(\left.y\right|x\right)=\frac{1}{Z\left(x\right)}exp(\sum_{t,k}\;\lambda_{k}t_{k}\left(y_{i-1},y_{i},x,i\right)+\sum_{i,l}\;\mu_{l}s_{l}\left(y_{i},x,i\right))$$ (3)

***Z***(***x***) is the normalization factor, represented by Eq (4):

$$Z\left(x\right)=\sum_{Y}\;exp(\sum_{i,k}\;\lambda_{k}t_{k}\left(y_{i-1},y_{i},x,i\right)+\sum_{i,l}\;\mu_{l}s_{l}\left(y_{i},x,i\right))$$ (4)

In the CRF model, the involved conditional probabilities are all probabilities obtained after normalization. In Eq (3), the term denoted by "unknown" represents features along with their respective weights. In the context of English discourse analysis, the formulation of these features is intricately tied to the templates used for feature extraction. Methods employed for weight learning encompass gradient descent, Newton method, quasi-Newton methods, and enhanced iterative scaling methods. Typically, the Viterbi algorithm is utilized for decoding to generate the output sequence Y. CRF faces significant drawbacks such as high training costs, complexity, and the necessity for manual construction of feature functions, which are pivotal in the CRF model. Consequently, the current trend is to use neural networks to extract features.

### The CRF-based discourse analysis algorithm

The preceding section offers a comprehensive introduction to CRF, particularly focusing on the basic concept of Linear-CRF. In this section, the learning algorithm and prediction algorithm of CRF used in this study are introduced. The Bidirectional Encoder Representations from Transformers (BERT) model is a pre-trained language model built on Transformer architecture, renowned for its bidirectional encoder capability. This feature enables BERT to effectively capture semantic nuances and contextual information within sentences, thereby generating robust and precise feature representations. BERT, being a versatile pre-trained model, demonstrates high performance across diverse NLP tasks such as semantic comprehension, text classification, and sequence labeling. Integrating BERT for feature vector generation enhances model versatility and adaptability, yielding superior outcomes across various tasks. This approach facilitates end-to-end learning by inputting text directly into BERT to obtain high-dimensional feature representations without the need for manual feature extractor design. Consequently, this streamlines model development and maximizes the language representation capabilities inherent in the BERT model. Thus, this study adopts the BERT model to generate feature vectors, transforming local feature functions into global counterparts by aggregating identical features across positions. This formulation allows CRF to express itself in terms of the inner product between weight and feature vectors [17–20].

Let the number of transfer features and state features be ***K***_**1**_ and ***K***_**2**_, and ***K* = *K***_**1**_ **+ *K***_**2**_, as follows:

$$f_{k}\left(y_{i-1},y_{i},x,i\right)=\left\{\begin{array}{l} t_{k}\left(y_{i-1},y_{i},x,i\right),k=1,2,\cdots ,K_{1}\\ S_{l}\left(y_{i},x,i\right),k=K_{1}+l;l=1,2,\cdots ,K_{2} \end{array}\right.$$ (5)

The transfer and state features at each position are summed:

$$f_{k}\left(y,x\right)=\sum_{i=1}^{n}\;f_{k}\left(y_{i-1},y_{i},x,i\right),k=1,2,\ldots ,K$$ (6)

The weight matrix ***w***_***k***_ is used to represent the weights of ***f***_***k***_ (***y***, ***x***):

$$w_{k}=\left\{\begin{array}{l} \lambda_{k},k=1,2,\ldots ,K_{1}\\ \mu_{l},k=K_{1}+l;l=1,2,\ldots ,K_{2} \end{array}\right.$$ (7)

Simplified as

$$P\left(y\left|x\right.\right)=\frac{1}{Z\left(x\right)}\;exp\;\sum_{k=1}^{K}\;w_{k}f_{k}\left(y,x\right)$$ (8)

The expression of ***Z***(***x***) reads:

$$Z\left(x\right)=\sum_{y}\;exp\;\sum_{k=1}^{K}\;w_{k}f_{k}\left(y,x\right)$$ (9)

In this way, the learning objective is transformed into finding model parameters ***Z***(***x***), and AdamW optimization algorithm is employed to update the BERT model iteratively [21]. Before learning, the logarithmic maximum likelihood function is given:

$$L\left(w\right)=log\;\prod_{x,y}\;P_{w}{\left(y\left|x\right.\right)}^{\tilde{p}\left(x,y\right)}=\sum_{x,y}\;\tilde{p}\left(x,y\right)\;log\;P_{w}\left(y\left|x\right.\right)$$ (10)

$\sum_{x,y}\;\tilde{p}\left(x,y\right)$ is an empirical probability distribution, which can be obtained from the training set. The model uses ***f***(***w***) = −***L***(***w***) as the loss function, and utilizes the AdamW optimization algorithm to find the function’s minimum value [22]. The AdamW algorithm is an improvement of gradient descent [23]. A gradient is a vector that increases the value of the directional function along this vector and decreases the fastest along the inverse of the vector. For unary functions, the gradient is derived by derivation; For multivariate functions, gradients are combined after partial derivation of each element separately [24].

The gradient descent problem can be expressed as follows. It can be assumed that ***f***(***x***) is a function with a continuous first-order partial derivative on ***R***^***n***^, the problem of minimizing ***minf***(***x***) can be addressed through an iterative process. This involves selecting an appropriate initial value ***x***^(**0**)^ and iteratively updating ***x*** according to the learning rate and gradient until the function converges. The iteration value of step ***k*** is presented as:

$$x^{\left(k\right)}\leftarrow x^{\left(k-1\right)}+\lambda_{k-1}p_{k-1}$$ (11)

***p***_***k*−1**_ is the gradient’s opposite, that is:

$$p_{k-1}=-\nabla f\left(x^{\left(k-1\right)}\right)$$ (12)

***λ***_***k*−1**_ is the learning rate, which is a hyperparameter, and the final result reads:

$$f\left(x^{\left(k\right)}+\lambda_{k}p_{k}\right)=minf\left(x^{\left(k\right)}+\lambda_{k}p_{k}\right)$$ (13)

### The Rasch model

This study introduces the Rasch model to estimate the difficulty of English discourse topics. The model exhibits stable cross-group and cross-context characteristics, addressing the limitation of relying solely on pass rates as indicators of difficulty within classical measurement theory. The fundamental principle of the Rasch model asserts that the probability of a participant answering a question correctly is a straightforward function of their individual ability ***θ*** and the question’s difficulty ***δ***. This functional relationship can be described as:

$$P_{ni1}=\frac{e^{\left(\theta_{n}-\delta_{i}\right)}}{1+e^{\left(\theta_{n}-\delta_{i}\right)}}$$ (14)

According to the Rasch model, when the student’s ability ***θ***_***n***_ is equal to the question’s difficulty ***δ***_***i***_, ***θ***_***n***_ − ***δ***_***i***_ = **0**, and ***P***_***ni*1**_ = **0.5**, that is, the probability of a correct answer is 50%. When ***θ***_***n***_ is higher than the ***δ***_***i***_, that is, ***θ***_***n***_ − ***δ***_***i***_ > **0**, the probability of correct answer is greater than 50%. Moreover, when the ***θ***_***n***_ is lower than the ***δ***_***i***_, that is, ***θ***_***n***_ − ***δ***_***i***_ < **0**, the probability of correct answer is less than 50%.

In the Rasch model framework, question difficulties are centered around an average of 0, with a range from [-∞,+∞]. With 0 as the center, the difficulty of the questions is divided into 5 levels. Questions questions with difficulties between (3, 1) are termed difficult, (1, -1) as medium, and (-1, -3) as easy. Questions with a difficulty of 3 or higher, or less than -3, are classified as extremely difficult.

## The english discourse component syntactic analysis model using the CRF model

### Model architecture

**Fig 2:**
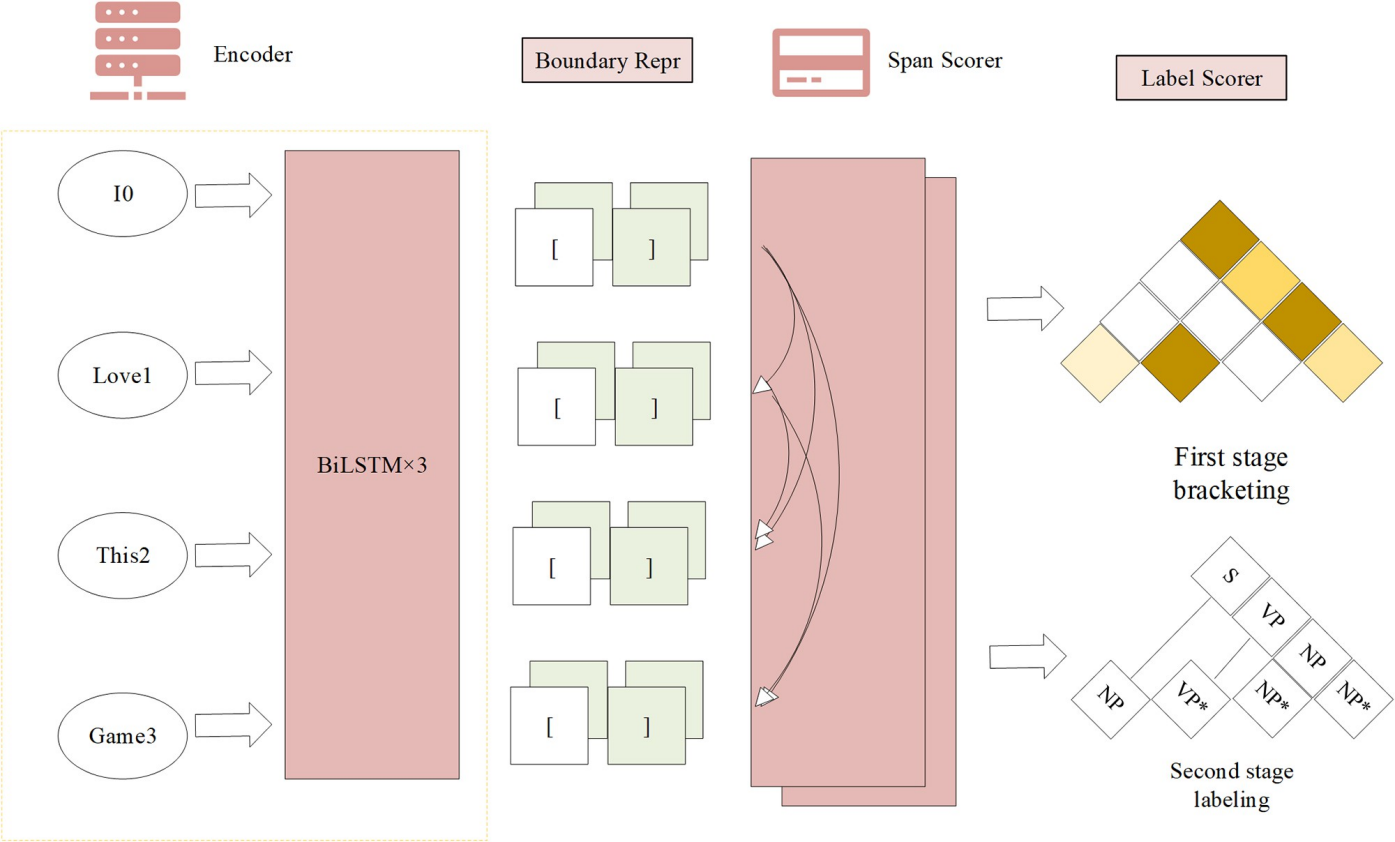
The architecture of the English discourse component syntactic analysis model.

The structure of the English discourse component syntactic analysis model by the CRF model is displayed in Fig 2.

Fig 2 is a specific model architecture constructed for syntactic analysis of English discourse components based on the CRF model in Fig 1. The Inside algorithm in CRF is an algorithm used to calculate the probability of all possible hidden state paths under a given observation sequence. The key to implementing the Inside algorithm on the Graphics Processing Unit (GPU) is to use its parallel computing capability to accelerate the processing of large-scale data and the execution of complex calculations. The Inside algorithm computes directly on the GPU using batching technology, addressing efficiency challenges in syntactic analysis. Introducing a second-order extension achieves results equivalent to the first-order Tree CRF [25]. A two-stage analytical approach, bracketing-then-labeling, is proposed, generating unmarked brackets initially and then assigning labels, proving more efficient and marginally superior to the first-order method. A novel scoring method based on block representation and an affine attention mechanism outperforms the minus-feature approach [26], significantly enhancing analytical performance by optimizing model parameters like Dropout.

**Fig 3:**
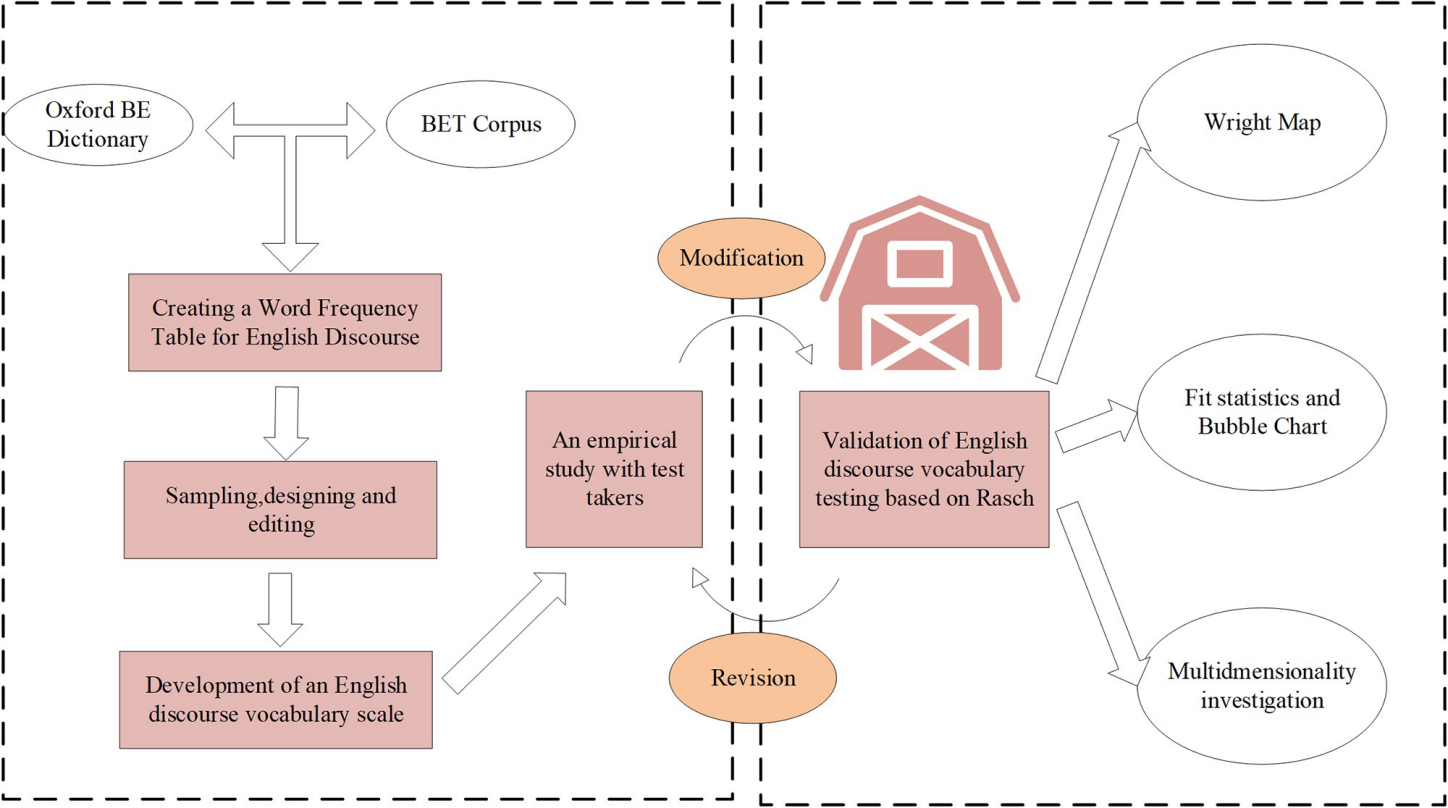
English discourse component syntactic analysis framework.

Individual abilities derived from the Rasch measurement model are selected as features alongside others for training and predicting the CRF model. For instance, in NLP, students’ reading comprehension scores from the Rasch model can serve as inputs for the CRF model, aiding tasks such as part-of-speech tagging or named entity recognition related to reading comprehension. Building on this theoretical foundation, the study provides preliminary validation by analyzing three aspects of Rasch. Firstly, it compares item difficulty and individual abilities on the same scale, offering insights into the overall test difficulty [27]. Secondly, it tests whether observed outcomes for each item align with Rasch model expectations using statistical data [28]. Hence, an English discourse component syntactic analysis framework is proposed, as indicated in Fig 3.

**Fig 4:**
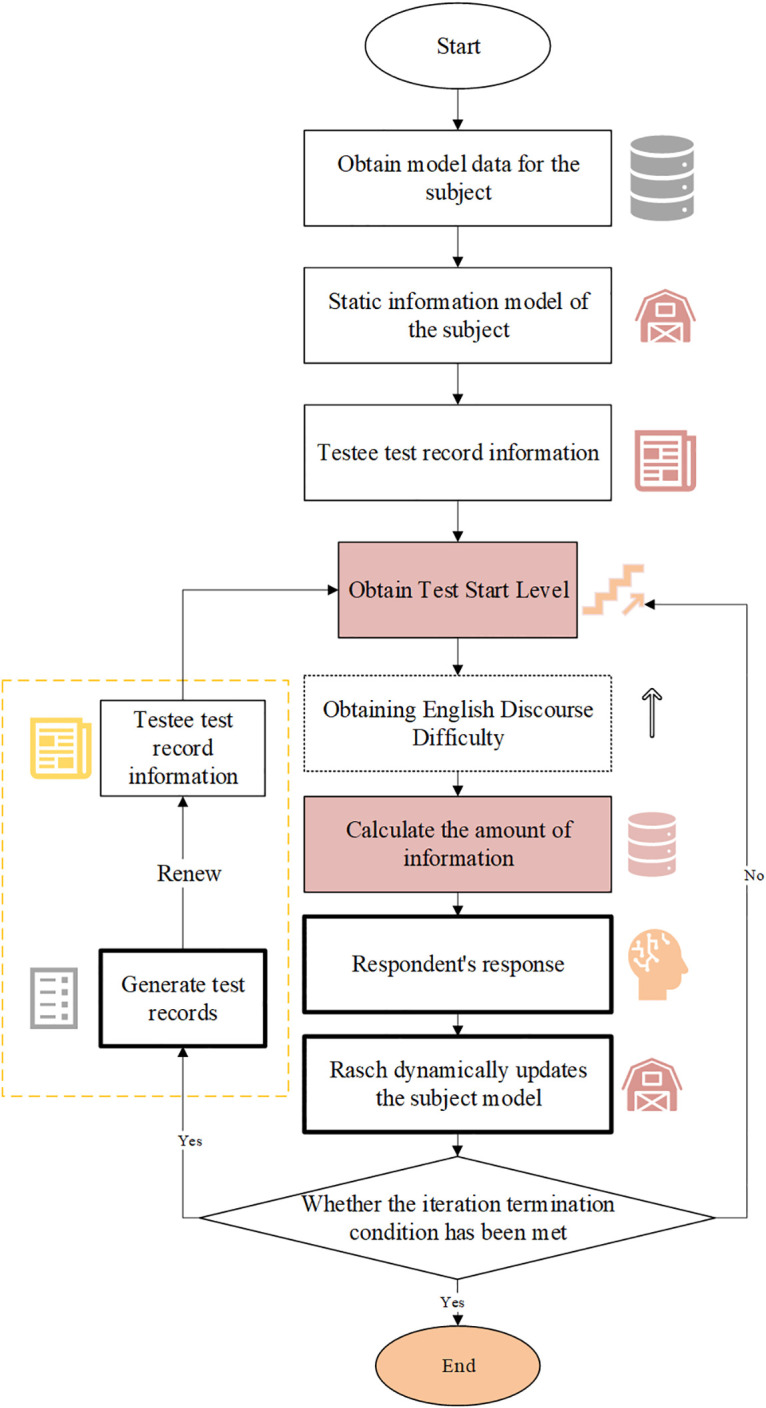
Flow chart of the Rasch-based test subject model.

Fig 3 illustrates the data selection process from dictionaries and the Business English Textbook (BET) for this study. An essential tool for testing English vocabulary is a word frequency table. The study utilizes a frequency list of 4,000 words compiled from an English discourse dictionary and word corpus for English learners, based on the Oxford University database [29]. Since the dictionary database lacks word frequency information, this study uses the frequency data from the BET word list, employing the Vertical Lookup (VLOOKUP) function to create a new English discourse word frequency list. Following the word sampling process, the study designs, edits, and revises the test format [30]. Furthermore, the application of the model is assessed across three dimensions to validate its effectiveness [31]. Firstly, it compares project difficulty against candidates’ abilities to determine if the test adequately measures varying participant skills. Secondly, statistical analysis explores whether test results for each item align with Rasch model estimates. Finally, Principal Component Analysis (PCA) of item residuals assesses whether the model structure is one-dimensional [32]. The flow chart of the test subject model by Rasch is drawn in Fig 4.

**Fig 5:**
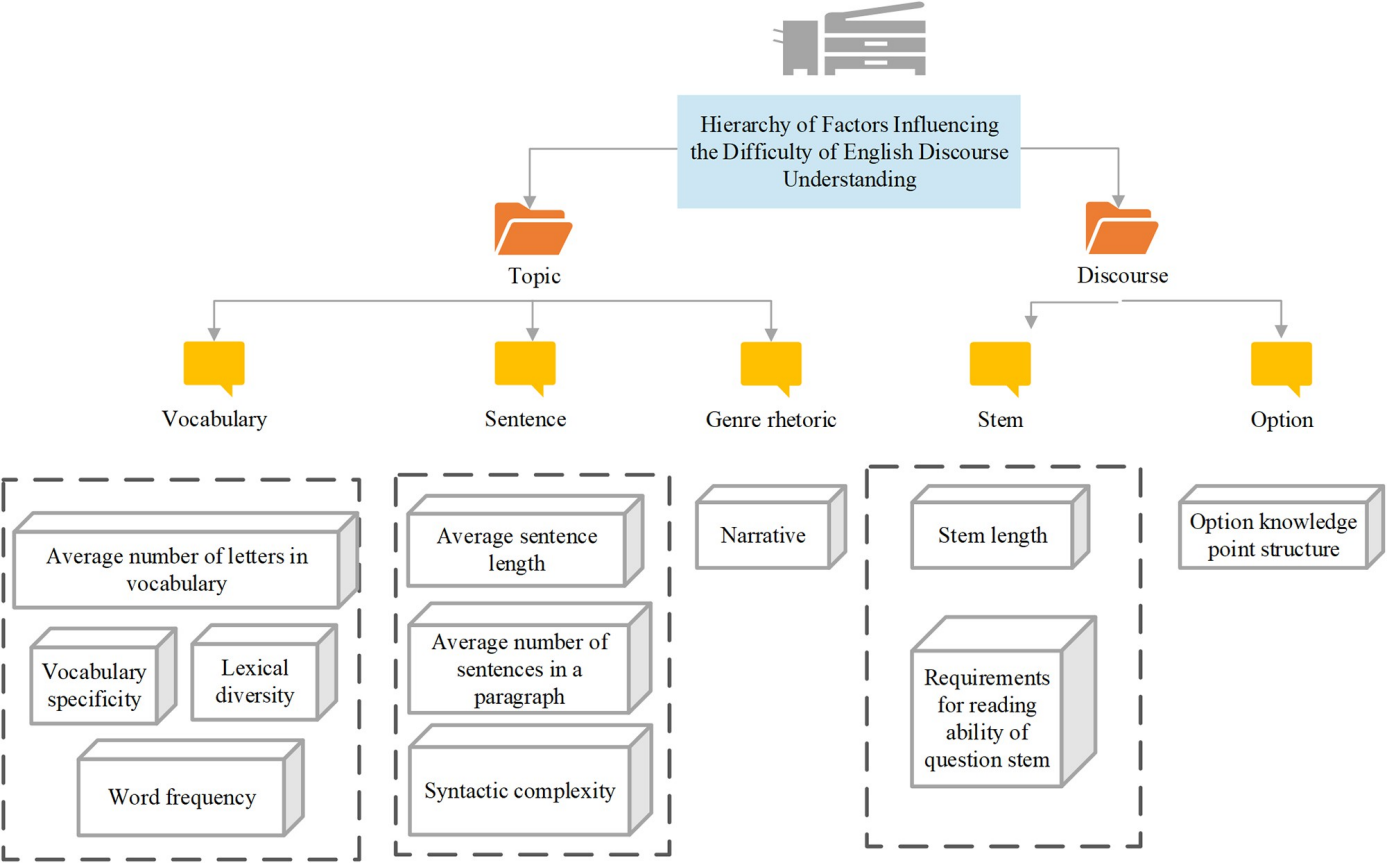
Hierarchical system of factors influencing the difficulty of objective questions in English discourse.

The subject module incorporates cross-module database calls to complete test response assessment of English reading comprehension based on the Rasch model and algorithm. The "subject" module constructs the initial subject model using static user data and historical answer data. It integrates the Rasch model within the adaptive test engine module for subject detection and ability level estimation [33–35]. This detection method continuously updates subject response data using the Rasch correlation algorithm, adjusting the subject model and generating current test records. It provides English discourse analysis ability results based on the subject’s test performance and updates test records accordingly. Fig 5 demonstrates a hierarchical system using analytic hierarchy principles to organize factors influencing the difficulty of objective questions in English discourse comprehension.

PCA allows the Rasch model to extract dimensions from the data’s first principal component, revealing its fundamental underlying structure. In an ideal scenario where the data is essentially one-dimensional and adheres to the Rasch model, residuals should exhibit no systematic relationship—indicating no discernible patterns or trends. Residuals in the Rasch model represent the difference between observed values and model-predicted values. When data fits the Rasch model, residuals should be randomly distributed without systematic patterns. This indicates that the model effectively captures data variation without missing essential information or including unnecessary noise. Examining systematic relationships in residuals helps assess how well the data conforms to the model, thus evaluating its fit and accuracy. The variance of standardized residuals is exhibited in Table 1.

**Table 1:** The variance of standardized residuals.

Residual variance component	Total raw variance in observations	Raw variance explained by measures	Raw unexplained variance (total)	Unexplained variance in 1st contrast
In Eigenvalue units	58.6	18.6	42	4.1
Percentage	100%	32.6%	68.5%	6.6%

The data in Table 1 provides detailed statistical information on residual variance using PCA and the Rasch model, which is crucial for assessing the model’s fit and explanatory power. The total residual variance is 58.6, representing the sum of variances of all residuals, and is used to measure the extent of variability in the observed data that the model fails to explain. The total raw variance of the observed data is 100%, corresponding to a value of 58.6, showing that this is the total variance of all observed data that the model attempts to explain. The raw variance explained by the model is 32.6%, corresponding to a value of 18.6, illustrating that the Rasch model can explain about one-third of the variance in the observed data, reflecting the model’s effective explanatory power of data variability. The raw variance not explained by the model is 68.5%, corresponding to a value of 42. It demonstrates that the model fails to explain about two-thirds of the variance in the observed data, reflecting the model’s limitations in capturing data variability. The unexplained variance in the first contrast is 6.6%, corresponding to a value of 4.1, indicating that the model fails to explain 6.6% of the variance in the first contrast. This further reveals the model’s differing explanatory abilities in various aspects. This comprehensive analysis of the Rasch model’s explanatory power shows that a high percentage of explained variance (such as 32.6%) indicates an effective explanation of data variability by the model. Moreover, the analysis of residual variance helps validate the model’s accuracy and reliability.

Experiments and Analysis: The experiments and analysis of dependency syntax analysis are conducted, encompassing three widely used datasets: English Penn Treebank (PTB), Chinese Treebank 7 (CTB7), and Conference on Natural Language Learning 2009 (CoNLL09). The data statistics of the component syntactic analysis dataset are outlined in Table 2.

**Table 2:** The data statistics of the component syntactic analysis dataset.

	Train	Test	Dev	Labels
PTB	38823	2622	1600	45
CoNLL09	22077	2555	1660	42
CTB7	46577	2799	4000	35

### Evaluation indicator

On the test data of the component syntactic analysis model, the results of different inference algorithms are usually evaluated by Precision (P), Recall (R), and F1-score (F1). These indicators can measure the model’s precision and recall in predicting the syntactic structure of components. Precision refers to the percentage of samples that the model predicts to be positive that is actually positive. It is expressed by Eq (15):

$$Precision=TP/\left(TP+FP\right)$$ (15)

***TP*** denotes the number of samples correctly predicted as positive by the model, while ***FP*** represents instances where the model incorrectly predicts negative cases as positive. Recall measures the proportion of positive samples correctly predicted by the model among all actual positive samples, defined as follows:

$$Recall=TP/\left(TP+FN\right)$$ (16)

***TP*** refers to true examples and ***FN*** represents false counterexamples. In other words, the samples in which the model incorrectly predicts positive examples as negative ones. F1-score is the harmonic mean of Precision and Recall, which considers the model’s precision and recall comprehensively, as plotted by Eq (17):

$$F1-score=2*\left(Precision*Recall\right)/\left(Precision+Recall\right)$$ (17)

The ***F*1** value is a comprehensive performance indicator, and if the model has high precision and recall, the ***F*1** value is also high. These indicators’ value range is between 0 and 1, and the closer to 1 means, the better the model performance. When evaluating a component syntactic analysis model’s performance, it is common to focus on these three indicators to understand how the model performs on different datasets.

### Experimental data design

In the second-order CRF model, the output dimension of the Multilayer Perceptron (MLP) layer for sibling features in dependency syntactic analysis and second-order features in component syntactic analysis is set to 100. The number of iterations for variational inference is set to 3. Parameter A, used to balance training losses between labeled and unlabeled trees in component syntactic analysis, is set to 0.1. Each syntactic analysis compares the four models: ***L***_***OC***_, ***C***_***RF***_, ***C***_***RF***_**2**_***O***_, and ***M***_***FVI***_. ***L***_***OC***_ represents a model that uses a local loss function; ***C***_***RF***_ and ***C***_***RF***_**2**_***O***_ refer to a CRF model that uses first- and second-order precise inference; ***M***_***FVI***_ indicates a model that uses mean field variational inference. All models employ pre-trained word vectors as inputs. 100-dimensional Glove word vectors are used for English data, and 100-dimensional Word2Vec word vectors are applied for Chinese data, which are trained on the GigaWord corpus. These experimental designs are chosen to improve the model’s performance on syntactic analysis tasks, improving the model’s input representation by effectively capturing semantic information about words and sentences.

## English discourse analysis by the Rasch model combined with CRF

### Performance evaluation of the English discourse component syntactic analysis model

**Fig 6:**
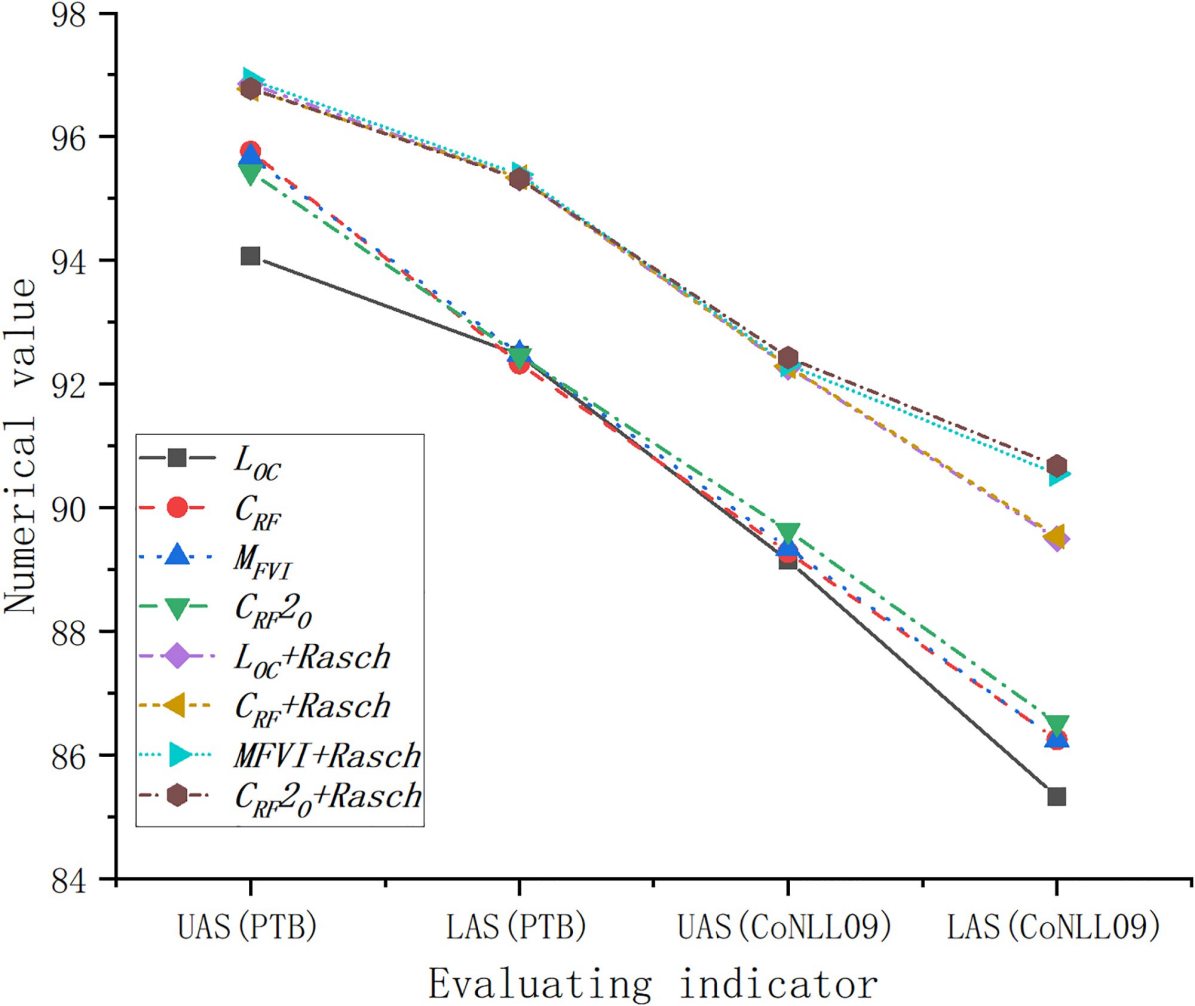
Performance evaluation of various inference algorithms on English PTB and CoNLL09 datasets.

To verify the English discourse component syntactic analysis model’s performance and test its practicability, this study takes the above dataset as the main training material, thus training and evaluating this model. The results of different inference algorithms of the proposed model on the English PTB and CoNLL09 datasets are illustrated in Fig 6.

In Fig 6, When the Rasch measurement model is not combined, the UAS of the four models ***L***_***OC***_, ***C***_***RF***_, ***C***_***RF***_**2**_***O***_, and ***M***_***FVI***_ obtained from the discourse component syntactic analysis model on the PTB dataset are 94.07, 95.76, 95.67, and 95.43, respectively. Four models’ LAS are 92.47, 92.33, 92.49, and 92.46, respectively. Combined with the Rasch measurement model, the UAS of ***L***_***OC***_, ***C***_***RF***_, ***C***_***RF***_**2**_***O***_, and ***M***_***FVI***_ models are 96.85, 96.77, 96.92, and 96.78. Moreover, the four models’ LAS are 95.33, 95.34, 95.39, and 95.32, respectively, presenting a good effect.

### Accuracy analysis of different algorithm models on PTB data sets

**Fig 7:**
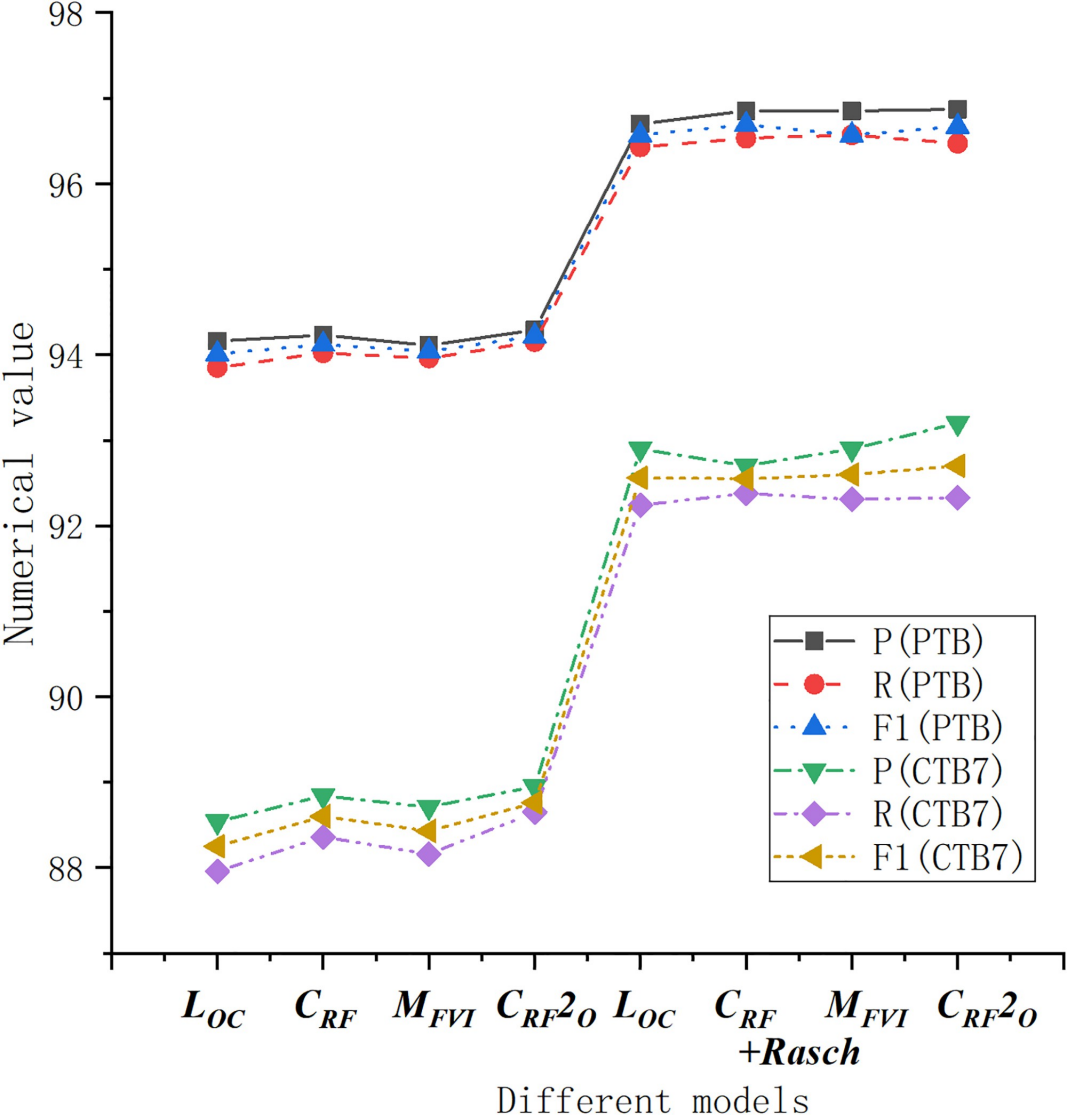
Precision and recall of diverse inference algorithms on PTB and CTB7 datasets.

Without the Rasch measurement model, different algorithm models’ precision and recall on English PTB and CTB7 datasets are suggested in Fig 7.

The left four datasets in Fig 7 display the accuracy (P), recall (R), and F1 scores of diverse inference algorithms on the English PTB and Chinese CTB7 datasets. For the English PTB dataset: The ***L***_***OC***_ model achieves an accuracy of 94.16, a recall of 93.85, and an F1 score of 94.01. The ***C***_***RF***_ model reaches an accuracy of 94.23, a recall of 94.02, and an F1 score of 94.12. The ***M***_***FVI***_ model attains an accuracy of 94.11, a recall of 93.96, and an F1 score of 94.04. The ***C***_***RF***_***2***_***O***_ model holds an accuracy of 94.29, a recall of 94.15, and an F1 score of 94.22. For the Chinese CTB7 dataset: The ***L***_***OC***_ model reaches an accuracy of 88.54, a recall of 87.96, and an F1 score of 88.25. The ***C***_***RF***_ model achieves an accuracy of 88.84, a recall of 88.36, and an F1 score of 88.60. The ***M***_***FVI***_ model has an accuracy of 88.71, a recall of 88.16, and an F1 score of 88.43. The ***C***_***RF***_***2***_***O***_ model attains an accuracy of 88.95, a recall of 88.65, and an F1 score of 88.76. In short, all models perform well on both datasets, with F1 scores ranging from 88.25 to 94.22. On both datasets, the ***C***_***RF***_***2***_***O***_ model has slightly higher accuracy, recall, and F1 scores compared to other models, but the performance differences between models are relatively small.

The right four datasets in Fig 7 represent the accuracy and recall metrics of various inference algorithms incorporating the Rasch model on the PTB and CTB7 datasets. For the English PTB dataset: The accuracy of the ***L***_***OC***_***+Rasch*** model is 96.7, with a recall of 96.43 and an F1 score of 96.57. The ***C***_***RF***_***2***_***O***_***+Rasch*** model achieves an accuracy of 96.85, a recall of 96.53, and an F1 score of 96.69. The ***M***_***FVI***_***+Rasch*** model attains an accuracy of 96.85, a recall of 96.57, and an F1 score of 96.57. The ***C***_***RF***_***2***_***O***_***+Rasch*** model has an accuracy of 96.87, a recall of 96.47, and an F1 score of 96.67. For the Chinese CTB7 dataset: The accuracy of the ***L***_***OC***_***+Rasch*** model is 92.9, with a recall of 92.24 and an F1 score of 92.56. The ***C***_***RF***_***2***_***O***_***+Rasch*** model achieves an accuracy of 92.7, a recall of 92.38, and an F1 score of 92.55. The ***M***_***FVI***_***+Rasch*** model demonstrates an accuracy of 92.9, a recall of 92.31, and an F1 score of 92.6. The ***C***_***RF***_***2***_***O***_***+Rasch*** model has an accuracy of 93.2, a recall of 92.33, and an F1 score of 92.7.

The data reveal that all models perform quite well on the English PTB and Chinese CTB7 datasets, with high levels of accuracy and recall. On the English PTB dataset, the ***C***_***RF***_***+Rasch*** model slightly outperforms other models in accuracy and F1 score, but the difference is not significant. On the Chinese CTB7 dataset, the ***C***_***RF***_***+Rasch*** model performs well in accuracy and recall, but other models’ performance is also very close. Overall, all models have achieved satisfactory results, demonstrating the effectiveness of the combined model in English discourse syntactic analysis tasks.

### Analysis of the superiority of the CRF model

To address the high cost of CRF training, parallel computing techniques are employed. These techniques involve distributing computational workloads across multiple processors or cores, thus accelerating the model training process. By parallelizing the training process, the time required to train the CRF model can be significantly reduced, making it more capable of handling larger datasets and more complex models. To demonstrate the CRF model’s superiority, this study experimentally compares the performance of the HMM, the Maximum Entropy Markov Model (MEMM), and CRF in sequence labeling tasks. The performance comparison results of the CRF model, HMM, and MEMM are provided in Table 3.

**Table 3:** Performance comparison results of HMM, MEMM, and CRF models.

Model	Precision	Recall	F1-score
HMM	0.85	0.70	0.77
MEMM	0.78	0.82	0.80
CRF	0.87	0.88	0.87

According to the data in Table 3, the CRF model achieves an accuracy of 0.87, remarkably outperforming the HMM’s 0.85 and the MEMM’s 0.78. In terms of recall and F1 score, the CRF model also excels, reaching 0.88 and 0.87, respectively, far surpassing the performance of the HMM and MEMM. This indicates that in the current sequence labeling tasks, the CRF model can more accurately capture dependencies and contextual information between sequences, leading to superior overall performance compared to the traditional HMM and MEMM. Firstly, unlike HMM which only considers local features and assumes independence between observations, the CRF model incorporates a rich set of features and global context, enhancing the accuracy of sequence prediction. Secondly, compared to MEMM, the CRF model directly models the dependencies between labels, avoiding the label bias problem and better generalizing to and handling complex sequence data.

## Conclusion

This study investigates the impact of integrating the Rasch measurement model with the CRF model to evaluate its effectiveness in English discourse analysis. One key feature of the Rasch measurement model is its provision of interpretable measurement outcomes. By incorporating this model, precise syntactic analysis results can be achieved, offering insights into sentence structure complexities and critical influencing factors. The following conclusions have been drawn from a comprehensive analysis and discussion of the experimental findings. (1) After adding the Rasch model, the four models ***L***_***OC***_, ***C***_***RF***_, ***C***_***RF***_**2**_***O***_, and ***M***_***FVI***_ obtained by the discourse component synthetic analysis model on the PTB database have UAS of 96.85, 96.77, 96.92, and 96.78. The four models have LAS of 95.33, 95.34, 95.39, and 95.32, respectively, demonstrating a good effect. (2) For the English PTB dataset: the ***L***_***OC***_***+Rasch***, ***C***_***RF***_***+Rasch***, ***M***_***FVI***_***+Rasch***, and ***C***_***RF***_***2***_***O***_***+Rasch*** models have a recall rate of 96.43, 96.53, 96.57, 96.47; These models have a precision of 96.7, 96.85, 96.85, 96.87; They have an F1 value of 96.57, 96.69, 96.57, and 96.67. For the CTB7 dataset: the ***L***_***OC***_***+Rasch***, ***C***_***RF***_***+Rasch***, ***M***_***FVI***_***+Rasch***, and ***C***_***RF***_***2***_***O***_***+Rasch*** models have a precision of 92.9, 92.7, 92.9, and 93.2; The above models have a recall of 92.24, 92.38, 92.31, and 92.33; They have an F1 value of 92.56, 92.55,92.6, and 92.7. It indicates that the performance of all models on the English PTB and CTB7 datasets is quite good, and the precision and recall have reached a high level.

Despite the significant achievements of this study, it is important to acknowledge its limitations and indicate directions for future work. Specifically, this study has the following limitations. Firstly, the dataset’s scale and diversity are vital factors that limit the comprehensive evaluation of the model’s performance. The current experiments are mainly based on standard datasets such as English PTB and CTB7. Although these datasets are widely recognized in the field, their scale is relatively small and may not fully cover the complexity and diversity of natural language. To more comprehensively validate the generalizability and robustness of the combined model, future research needs to expand the dataset’s scale and incorporate text data from more domains, styles, and language characteristics to ensure the model works effectively in a wider range of scenarios. Secondly, although this study has demonstrated the advantages of combining the Rasch measurement model and the CRF model in syntactic analysis through a series of experiments, it is still necessary to conduct more diversified experimental verifications and comparative analyses. This includes comparing with more advanced syntactic analysis models and examining performance under different evaluation metrics and task settings. Through these in-depth comparative analyses, this study can more accurately grasp the combined model’s strengths and weaknesses and provide a strong basis for further optimization.

Looking ahead, to further improve the performance of syntactic analysis, this study can consider the following directions. First, more semantic information and contextual features are introduced. Although the current model considers the text’s semantics and contextual information to some extent, there is still room for improvement. By incorporating more extensive semantic resources (like word vectors, dependency relations, etc.) and sophisticated contextual representations (including sentence-level, paragraph-level, and even discourse-level information), this study aims to enhance the model’s comprehension of textual nuances, thereby advancing the accuracy of syntactic analysis. Second, it is necessary to explore more complicated model combination methods. The current study adopts a relatively straightforward model combination approach. However, in the future, more complex combination strategies, such as multi-level fusion and dynamic weight adjustment, can be tried to more flexibly leverage the advantages of different models and achieve performance optimization. In conclusion, this study provides a beneficial exploration and preliminary results for the application of the Rasch measurement model and the CRF model in English discourse syntactic analysis. However, future efforts are still needed in dataset expansion, experimental validation, and model optimization to further advance the field.

## Supporting information

S1 Data (RAR)

## References

[1] Andrich D, Pedler P. A law of ordinal random error: The Rasch measurement model and random error distributions of ordinal assessments. Measurement. 2019; 131(2): 771–781.

[2] Sutton C, McCallum A. An introduction to conditional random fields. Foundations and Trends^®^ in Machine Learning. 2012; 4(4): 267–373.

[3] Jayanti D, Hidayat D N. Grammatical Cohesive Devices in Reading Text: A Discourse Analysis of English Test for Junior High School. JET ADI BUANA. 2021; 6(1):1–6.

[4] Wang S, Wang G, Qiang F U, Song Y, Liu J, Sheng H E. STABC-IR:An air target intention recognition method based on bidirectional gated recurrent unit and conditional random field with space-time attention mechanism. Journal of Aeronautics and Astronautics of China: English version. 2023, 36(3):19.

[5] Geng B. Text segmentation for patent claim simplification via Bidirectional Long-Short Term Memory and Conditional Random Field. Computational Intelligence. 2022; 38(1):205–215.

[6] Mokshein S E, Ishak H, Ahmad H. The use of rasch measurement model in English testing. Jurnal Cakrawala Pendidikan. 2019; 38(1): 16–32.

[7] Dewi H H, Damio S M, Sukarno S. Item analysis of reading comprehension questions for English proficiency test using Rasch model. Research and Evaluation in Education. 2023; 9(1): 24–36.

[8] Fleary S A, Freund K M, Nigg C R. Development and validation of assessments of adolescent health literacy: a Rasch measurement model approach. BMC public health. 2022; 22(1): 585. doi: 10.1186/s12889-022-12924-4 35331182 PMC8953064

[9] Wolfs Z G, Brand-Gruwel S, Boshuizen H P A. Assessing Tonal Abilities in Elementary School Children: Testing Reliability and Validity of the Implicit Tonal Ability Test Using Rasch Measurement Model. SAGE Open. 2023; 13(3): 21582440231199041.

[10] Roberts F, Aziz A A, Effendi M, Matore E M. Establishing the validity and reliability of the Malaysian English language textbook evaluation checklist (MELTEC) using Rasch measurement model (RRM). Journal of Language Teaching and Research. 2022; 13(1): 38–45.

[11] Li Y, Lai C, Feng J, Feng H. Chinese and English Elementary Discourse Units Recognition based on Bi-LSTM-CRF Model. 2020; 329–343.

[12] Aryadoust V, Ng L Y, Sayama H. A comprehensive review of Rasch measurement in language assessment: Recommendations and guidelines for research. Language Testing, 2021; 38(1): 6–40.

[13] Tian J, Wang B, Guo R, Wang Z, Cao K, Wang X. Adversarial attacks and defenses for deep-learning-based unmanned aerial vehicles. IEEE Internet of Things Journal. 2021; 9(22): 22399–22409.

[14] Sutton C, McCallum A. An introduction to conditional random fields. Foundations and Trends^®^ in Machine Learning. 2012; 4(4): 267–373.

[15] Li Y, Li C, Li X. A comprehensive review of Markov random field and conditional random field approaches in pathology image analysis. Archives of Computational Methods in Engineering. 2022; 29(1): 609–639.

[16] Li D, Zhang L. Exploring teacher scaffolding in a CLIL-framed EFL intensive reading class: A classroom discourse analysis approach. Language Teaching Research. 2022; 26(3):333–360.

[17] Envelope D L. Modes and intersemiotic cohesion in student presentations performed online: An SF-informed multimodal discourse analysis. English for Specific Purposes. 2023; 69(1):67–79.

[18] Math L, Fatima R. Efficient Vessel Segmentation Based on Proposed Adaptive Conditional Random Field Model. Recent advances in computer science and communications. 2022;2(5):15.

[19] Wang S, Wang G, Qiang F U. STABC-IR:An air target intention recognition method based on bidirectional gated recurrent unit and conditional random field with space-time attention mechanism. Journal of Aeronautics and Astronautics of China: English Edition. 2023; 24(3):3.

[20] Li J T, Tong F, Irby B J, Lara-Alecio R, River H. The effects of four instructional strategies on English learners’ English reading comprehension: A meta-analysis. Language Teaching Research. 2021;23(3):136

[21] Moon S, Lee G, Chi S. Automated system for construction specification review using natural language processing. Advanced Engineering Informatics. 2022; 51(4):101495-.

[22] Li R, Chen X. An efficient interactive multi-label segmentation tool for 2D and 3D medical images using fully connected conditional random field. Computer Methods and Programs in Biomedicine. 2022; 213(2):106534-. doi: 10.1016/j.cmpb.2021.106534 34839271

[23] Stolt M, Kottorp A, Suhonen R. The use and quality of reporting of Rasch analysis in nursing research: A methodological scoping review. International journal of nursing studies. 2022; 132(3):104244. doi: 10.1016/j.ijnurstu.2022.104244 35635906

[24] Lee S Y, Hong A J. Psychometric Investigation of the Cultural Intelligence Scale Using the Rasch Measurement Model in South Korea. Sustainability. 2021; 13(6):3139.

[25] Kieffer M J, Mancilla-Martinez J, Logan J K. Executive functions and English reading comprehension growth in Spanish-English bilingual adolescents. Journal of Applied Developmental Psychology. 2021; 73(3):101238.

[26] Trendler Günter. The incoherence of Rasch measurement: A critical comparison between measurement in psychology and physics. Personality and Individual Differences. 2022; 189:111408-.

[27] Valizadeh M. Shanlax International Journal of Education s h a n l a x The Effect of Reading Comprehension Strategies Instruction on EFL Learners’ Reading Anxiety Level. Shanlax International Journal of Education. 2021; 9(1):53–58.

[28] Motta C D, Célia B. Carvalho, Pato M T. Rasch Measurement of the Brief Situational Test of Emotional Management in a Large Portuguese Sample. Journal of Psychoeducational Assessment. 2021; 39(1):112–127.

[29] Ma C, Zhou S, Chi J. Seismic performance analysis of underground structures based on random field model of soil mechanical parameters. Progress in Earthquake Research (in English). 2022; 2(4):3–10.

[30] Mccarron S P, Kuperman V. Effects of year of post-secondary study on reading skills for L1 and L2 speakers of English. Journal of Research in Reading. 2022; 45(1):43–64.

[31] Nguyen K V, Nguyen N D, Do N T. ViReader: A Wikipedia-based Vietnamese reading comprehension system using transfer learning. Journal of Intelligent & Fuzzy Systems: Applications in Engineering and Technology. 2021;2(1):41.

[32] Rostadmo M, Strmme S L, Nylenna M. How well do doctors understand a scientific article in English when it is not their first language? A randomised controlled trial. BMJ Open. 2021;2(6):2–10. 10.1136/bmjopen-2020-043444 PMC8194323 34112640

[33] Alenezi S. Investigating Saudi EFL Students’ Knowledge and Beliefs Related to English Reading Comprehension. Arab World English Journal. 2021; 12(1):339–356.

[34] Hu L J. On the Reconstruction of Teachers’Role of Business English Reading Classroom Teaching Based on Literature Circle. Sino American English Teaching: English Version. 2023; 20(3):90–96.

[35] Mccarron S P, Kuperman V. Effects of year of post-secondary study on reading skills for L1 and L2 speakers of English. Journal of Research in Reading. 2022; 45(1):43–64.

